# Protective Role of Hesperidin Against Deltamethrin-Induced Cardiovascular Structural Damage: Involvement of Caspase-3-Driven Apoptosis and Fibrosis Suppression in Rats

**DOI:** 10.3390/jcdd13040159

**Published:** 2026-04-03

**Authors:** Burcu Gültekin, Halime Tuba Canbaz, Hasan Basri Savaş, Gökhan Cüce, Sabiha Serpil Kalkan

**Affiliations:** 1 Department of Histology and Embryology, Faculty of Medicine, Necmettin Erbakan University, Konya 42090, Türkiye; gcuce@erbakan.edu.tr (G.C.); skalkan@erbakan.edu.tr (S.S.K.); 2 Department of Histology and Embryology, Hamidiye Faculty of Medicine, Health Sciences University, Istanbul 34668, Türkiye; halimetuba.canbaz@sbu.edu.tr; 3 Department of Medical Biochemistry, Faculty of Medicine, Mardin Artuklu University, Mardin 47000, Türkiye; hasansavas@artuklu.edu.tr

**Keywords:** aorta, apoptosis, cardiotoxicity, deltamethrin, fibrosis, hesperidin

## Abstract

Background and Objectives: Deltamethrin (DLM), a widely used pyrethroid insecticide, has been linked to cardiotoxic effects in non-target organisms. Hesperidin (HSP), a dietary bioflavonoid with antioxidant and cardioprotective properties, may counteract these effects. This study investigated the protective role of HSP against DLM-induced cardiotoxicity in male Wistar Albino rats. Materials and Methods: Thirty-two rats were divided into four groups: Control, DLM, DLM + HSP 100, and DLM + HSP 300. At the end of the experiment, serum ischemia-modified albumin (IMA), glucose, cholesterol, triglyceride, and HDL levels were analyzed. Cardiac and aortic tissues were assessed histopathologically. Masson’s trichrome staining evaluated cardiac fibrosis, Verhoeff–Van Gieson staining examined elastin and tunica media thickness, and caspase-3 expression in the aorta was determined immunohistochemically. Results: DLM administration caused cardiac and aortic damage by increasing IMA, glucose, caspase 3 activities, and tunica media thickness. HSP treatment, particularly at 300 mg/kg, reduced IMA (0.28 ± 0.02 vs. 0.60 ± 0.03 AU), glucose (141.12 ± 11.70 vs. 207.06 ± 9.85 mg/dL), cardiac histopathological damage score (2.17 ± 0.41 vs. 9.02 ± 1.35), tunica media thickness (95.29 ± 4.29 vs. 114.95 ± 17.20 µm), and caspase-3 expression score (0.62 ± 0.74 vs. 2.87 ± 0.35). All results showed significance at the *p* < 0.05 level. Conclusions: HSP exhibited dose-dependent protective effects against DLM-induced oxidative stress, apoptosis, and cardiovascular injury, suggesting its potential as a therapeutic candidate against pesticide-related cardiotoxicity.

## 1. Introduction

The release of pesticides into the environment poses a serious threat to public health [1]. Humans are exposed to these chemicals both directly through occupational contact and indirectly via the consumption of food and water contaminated with toxic compounds [2]. The cardiovascular system is considered particularly vulnerable to the harmful effects of various pesticides [3]. Recent studies have reported a significant association between the consumption of pesticide-contaminated fruits and vegetables and an increased risk of coronary heart disease [4]. In addition, Feriani et al., 2016 identified pesticide exposure as a risk factor for atherosclerotic vascular diseases [5]. Several mechanisms have been proposed to explain the atherogenic effects of these pollutants, among which pesticide-induced oxidative stress and inflammation are thought to play critical roles in the initiation and progression of vascular events. Today, the relationship between pesticide-induced alterations in lipid and lipoprotein profiles and vascular arterial dysfunction is well established [6].

Deltamethrin (DLM) [S-alpha-cyano-3-phenoxybenzyl-(1R)-cis-3-(2,2-dibromovinyl)-2,2-dimethylcyclopropane carboxylate] is a type II synthetic pyrethroid widely used worldwide as an insecticide and acaricide [7]. It is also applied in urban green areas and residential settings for controlling garden pests, as well as topically in livestock to prevent vector-borne diseases transmitted by ticks, mites, fleas, and flies. Although Deltamethrin (DLM) is often preferred due to its rapid metabolism and relatively low toxicity profile in mammals [8], it still poses significant hazards as an environmental and industrial contaminant. Dose-dependent toxic effects have been documented not only in mammals but also particularly in birds and fish. Beyond its hepatotoxic, nephrotoxic, allergenic, and immunosuppressive effects, DLM has also been reported to exert toxicity on the cardiovascular and reproductive systems [9]. Carotid intima–media thickness (IMT) is a well-recognized indicator of atherosclerosis and is associated with both current and future risks of coronary heart disease and stroke [10]. The lipophilic nature of DLM facilitates its diffusion across cell membranes, leading to the excessive production and accumulation of reactive oxygen species (ROS) [11]. This oxidative damage contributes to the development of various cellular injuries by inducing lipid peroxidation, DNA damage, and protein degradation [12,13,14].

Hesperidin (HSP; 3,5,9-trihydroxy-4′-methoxy-7-o-rutinosyl flavanone) is a biologically active flavanone belonging to the flavonoid class. It is predominantly found in citrus fruits such as oranges, lemons, and grapefruits. Numerous studies have demonstrated the wide-ranging health benefits of HSP, from cancer prevention to the alleviation of menopausal symptoms. Its anti-allergic, anticancer, antioxidant, and anti-inflammatory effects are well documented in the literature. The antioxidant activity of Hesperidin (HSP) occurs through direct scavenging of free radicals as well as enhancement of the endogenous antioxidant defense system. In this way, it protects tissues against DNA and protein damage caused by both internal factors (e.g., oncogenes) and external factors (e.g., radiation, inflammation, toxins) [15]. Within the cardiovascular system, flavonoids have been shown to mediate endothelium-dependent and/or independent vasodilation, prevent endothelial dysfunction, inhibit platelet aggregation, suppress smooth muscle cell proliferation, and reduce oxidative stress and inflammation. Through these properties, flavonoids hold promise as anti-obesity, anti-diabetic, and anti-atherosclerotic agents [16].

Although numerous studies have addressed the cardiovascular toxicities of pesticides, the role of DLM in the development of atherosclerosis and the oxidative stress-mediated mechanisms underlying this process remain insufficiently clarified. Furthermore, the protective potential of HSP, a potent natural flavonoid, against DLM-induced cardiovascular injury has not been adequately investigated. This study is therefore novel in its aim to contribute to the understanding of pesticide-induced vascular injury while exploring the potential protective effects of HSP. The objective of this research was to evaluate the adverse effects of DLM exposure on the cardiovascular system in rats and to investigate the possible protective role of HSP against this toxicity. To achieve this aim, experimental animals were divided into control, DLM, DLM + HSP100, and DLM + HSP300 groups. Cardiovascular injury markers, including carotid IMT, ischemia-modified albumin (IMA), and oxidative stress parameters, were assessed to comparatively analyze the cardiotoxic effects of DLM and the potential protective role of HSP.

## 2. Materials and Methods

### 2.1. Experimental Animals

A total of 32 male Wistar Albino rats, weighing 250–300 g and aged 16–18 weeks, were used in this study. All experimental procedures involving animals were carried out in accordance with the ARRIVE guidelines and the Guide for the Care and Use of Laboratory Animals, and received prior approval from the Local Ethics Committee of Necmettin Erbakan University (Approval No: 2022-035; approved on 6 July 2022). The rats were housed in standard cages with sawdust-covered floors, wire lids, integrated water bottles, and feeding compartments, with four rats per cage (eight cages in total). Standard pellet chow, commonly used in scientific research and providing balanced nutrition according to the animals’ age, weight, and physiological needs, was supplied. Food and water were available ad libitum. Environmental conditions were maintained at 24 ± 1 °C, 45 ± 5% humidity, and a 12-h light/12-h dark cycle. All animals were weighed at the beginning and end of the experiment. No analgesic or anti-inflammatory agents were administered during the experimental period to avoid potential interference with the biochemical and histopathological outcomes. At the end of the study, anesthesia was administered (i.p.) using a combination of Ketamine HCl (50 mg/kg) and Xylazine HCl (10 mg/kg) to minimize pain. Blood samples were collected via cardiac puncture, transferred into anticoagulant and gel-containing biochemistry tubes, and used for routine biochemical analysis as well as for the assessment of oxidative stress and antioxidant balance parameters in serum.

### 2.2. Experimental Design

All solutions were freshly prepared before each administration. DLM (CAS No: 52918-63-5; Supelco^®^, Sigma-Aldrich, Darmstadt, Germany) and HSP (CAS No: 520-26-3; Supelco^®^, Sigma-Aldrich, Darmstadt, Germany) were used in the study. According to the literature, the oral LD_50_ of DLM in rats is 128 mg/kg [17]. Therefore, a dose of 1.28 mg/kg DLM (equivalent to 1/100 of LD_50_) was selected. This sublethal dose has previously been reported to induce biochemical and histopathological alterations without causing mortality and is widely accepted as a safe reference dose for sublethal toxicity studies. Although this dose is higher than typical environmental exposure levels, it may represent chronic or cumulative exposure scenarios for humans and non-target organisms in the food chain, particularly considering bioaccumulation and repeated low-dose exposure. While the selected DLM dose is sublethal in rats, potential exposure to other organisms in the food chain should also be considered, as some species may be more sensitive to deltamethrin, and bioaccumulation could result in higher internal concentrations in predators [18]. For HSP administration, dosage ranges defined by Jia et al., 2022 (100 mg/kg as low dose, 300 mg/kg as high dose) were adopted [19]. Although Jia et al. classified 300 mg/kg as a high dose, Li et al., 2019 demonstrated that even 500 mg/kg of HSP caused no significant alterations in urinary parameters, hematological and biochemical values, organ weights, pathological findings, or food intake [20]. Therefore, the doses of 100 and 300 mg/kg, supported by previous literature as both effective and safe, were selected for this study. HSP was administered 30 min after deltamethrin DLM exposure to evaluate its therapeutic/reversal effects on established cardiovascular damage. HSP was applied at two different doses (100 and 300 mg/kg) to assess potential dose-dependent effects and treatment efficacy. This timing was selected to avoid direct interactions between HSP and DLM, which could interfere with DLM absorption, distribution, or metabolism and thus confound the interpretation of its toxic effects. The 32 rats were randomly assigned into four groups (*n* = 8 per group) and treated for 30 consecutive days:

Control group (*n* = 8): 1 mL corn oil orally (gavage) administered daily.

DLM group (*n* = 8): 1.28 mg/kg DLM dissolved in 1 mL corn oil, administered orally (gavage) daily.

DLM + HSP 100 group (*n* = 8): DLM (1.28 mg/kg in 1 mL corn oil) followed 30 min later by HSP (100 mg/kg dissolved in distilled water), administered orally (gavage).

DLM + HSP 300 group (*n* = 8): DLM (1.28 mg/kg in 1 mL corn oil) followed 30 min later by HSP (300 mg/kg dissolved in distilled water), administered orally (gavage).

### 2.3. Biochemical Analysis

At the end of the experiment, blood samples collected in gel-containing biochemistry tubes were centrifuged at 1500 *g* for 10 min to obtain serum. Supernatants were aliquoted into Eppendorf tubes, labeled, and stored at −80 °C until analysis. Before analysis, all samples were thawed simultaneously at room temperature, vortexed, and prepared for biochemical assays. Serum IMA levels were determined using the albumin cobalt-binding test. Briefly, 95 μL of serum was incubated with 5 μL cobalt chloride (final concentration 0.58 mmol/L) for 5 min. Following incubation, 25 μL dithiothreitol (final concentration 1.67 mmol/L) was added to detect unbound cobalt. Absorbance was measured spectrophotometrically at 500 nm. A five-point calibration curve (5–180 U/mL) was used to calculate IMA concentrations [21,22]. Serum cholesterol (CHOL), triglyceride (TG), HDL, and glucose levels were determined spectrophotometrically using the modified Erel method with a commercial kit on an automated biochemical analyzer (AU5800; Beckman Coulter, Brea, CA, USA) [23].

### 2.4. Histological Examinations

After blood collection, the animals were sacrificed, and cardiac and aortic tissues were dissected and fixed in 10% formalin for microscopic evaluation. For deparaffinization, sections were immersed in xylene for three consecutive 20-min intervals, followed by rehydration through a graded ethanol series from absolute ethanol to 50%. Subsequently, the sections were stained with hematoxylin and eosin (H&E). After staining, the slides were dehydrated through an ascending ethanol series (from 50% to absolute ethanol in 10-min increments) and mounted with coverslips using Entellan.

All histopathological and immunohistochemical evaluations were performed with concealed group allocation through random coding of the samples. The coding process was carried out by a researcher not involved in the evaluation procedures. Histopathological scoring and immunohistochemical assessments were independently conducted by two blinded observers according to the same predefined criteria. Measurements of tunica media thickness were performed on Verhoeff–Van Gieson-stained sections by a single experienced blinded observer to ensure standardization of the measurement protocol.

For each animal, six randomly selected fields per staining were evaluated, and animal-based mean values were used for statistical analysis. Histopathological analyses were carried out using a Zeiss (Carl Zeiss Microscopy GmbH, Jena, Germany) Lab.A1 light microscope equipped with a Zeiss Axicam ERc 5s camera, and evaluations were conducted through a double-blind histopathological assessment.

#### 2.4.1. Cardiac Tissue (H&E and Masson’s Trichrome Staining)

Cardiac analyses were performed on left ventricular myocardial sections. The sections were deparaffinized, rehydrated through graded alcohols, and stained with H&E for structural evaluation based on four distinct parameters, including congestion, edema, inflammatory cell infiltration, and vacuolization. Each parameter was scored semi-quantitatively (0 = absent, 1 = mild, 2 = moderate, 3 = severe). The scores for each of the four parameters, which ranged from 0 to 3, were summed to obtain a total histological score ranging from 0 to 12 [24]. For fibrosis assessment, sections were stained using a commercial Masson’s trichrome kit (PLKit20-150, Histomed, Ankara, Turkey), where collagen fibers appeared blue. Random fields around vessels were analyzed for collagen deposition [25].

#### 2.4.2. Aortic Tissue (H&E and Verhoeff–Van Gieson Staining)

The histopathological and immunohistochemical evaluations of the aorta were performed through a detailed examination of the tunica intima and tunica media layers in sections obtained from a consistent region of the thoracic aorta. Aortic sections stained with H&E were examined for endothelial damage, intimal detachment, and vacuolization [26]. Elastic fibers and tunica media thickness were evaluated using Verhoeff–Van Gieson staining (GBL Kit, Istanbul, Turkey). Tunica media thickness was measured at six random sites per section at 40× magnification using Zen Blue 3.4 software (Carl Zeiss, Jena, Germany), and mean values were calculated for each group [27].

#### 2.4.3. Immunohistochemistry (Caspase-3 Expression in Aortic Tissue)

Paraffin sections (4 μm) were mounted on poly-L-lysine–coated slides. After deparaffinization and antigen retrieval, endogenous peroxidase activity was blocked with 3% hydrogen peroxide. Non-specific binding was minimized using a blocking solution (Super Block, ScyTek, Logan, UT 84321, USA). Sections were incubated overnight with primary anti-caspase-3 antibody (AB184787, Abcam, Cambridge, CB2 0AX, UK), followed by secondary antibody and streptavidin–peroxidase treatment. Visualization was achieved using 3,3′-diaminobenzidine (DAB), and counterstaining was performed with Mayer’s hematoxylin. All slides were examined under a Zeiss Lab.A1 light microscope, and images were captured using a Zeiss AxioCam ERc 5s camera. Immunostaining was semi-quantitatively evaluated based solely on staining intensity and scored as follows: 0 (negative), 1 (weak), 2 (moderate), and 3 (strong). For the semi-quantitative analysis of Caspase-3 immunoreactivity, six microscopic fields were randomly selected from each section, and the mean staining score was calculated per preparate. To minimize subjective bias, the scoring process was performed by two independent histologists who were blinded to the experimental groups [28,29].

### 2.5. Statistical Analysis

Data normality was assessed using the Shapiro–Wilk test, confirming a normal distribution. Differences between groups were analyzed using one-way analysis of variance (ANOVA). When significant differences were detected, Tukey’s post hoc test was applied for pairwise comparisons. Data are presented as mean ± standard deviation (SD) (*n* = 8 for each group). A *p*-value < 0.05 was considered statistically significant. Statistical analyses were performed using GraphPad Prism 8 software (GraphPad Software, Boston, MA, USA), while Microsoft Office 365 (Microsoft Corp., Redmond, WA, USA) was used for data management and additional calculations.

## 3. Results

### 3.1. Biochemical Analyses

**Figure 1:**
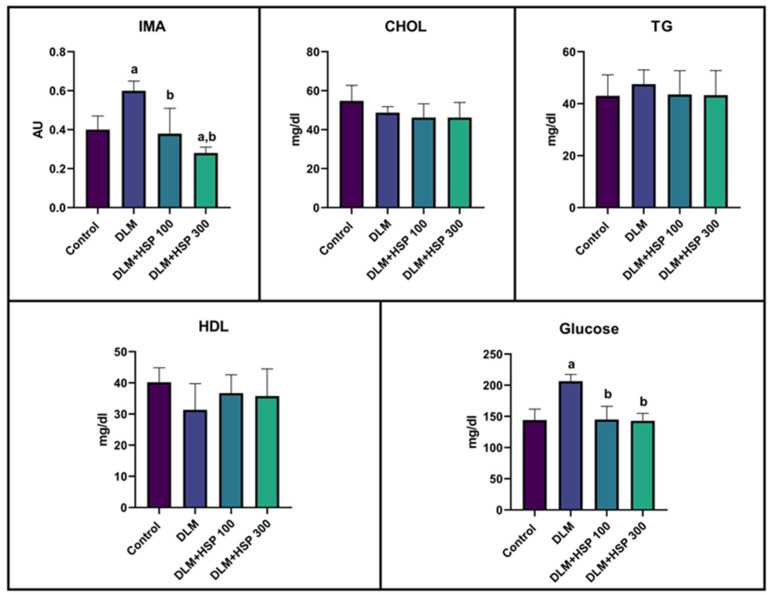
Effects of DLM and HSP administration on serum biochemical parameters. Levels of ischemia-modified albumin (IMA), cholesterol (CHOL), triglycerides (TG), high-density lipoprotein (HDL), and glucose were measured. Data are presented as mean ± SD (*n* = 8 per group). Statistical significance is indicated by a (*p* < 0.05 vs. Control group) and b (*p* < 0.05 vs. DLM group).

Biochemical parameters, including IMA, total CHOL, TG, HDL, and glucose, were evaluated across all experimental groups. No statistically significant differences were observed among the groups with respect to CHOL, TG, and HDL levels. Notably, IMA, a marker of oxidative stress, was significantly elevated in the DLM-treated group compared to the Control group. In contrast, IMA levels in the low-dose HSP treatment group were comparable to those of the Control group, whereas the high-dose HSP group exhibited IMA levels even lower than the Control. Elevated glucose levels observed in the DLM group were effectively restored to baseline following both low- and high-dose HSP treatments (Figure 1).

### 3.2. Histological Analyses

#### 3.2.1. Hematoxylin–Eosin (H&E) and Masson’s Trichrome Staining of Cardiac Tissue

**Figure 2:**
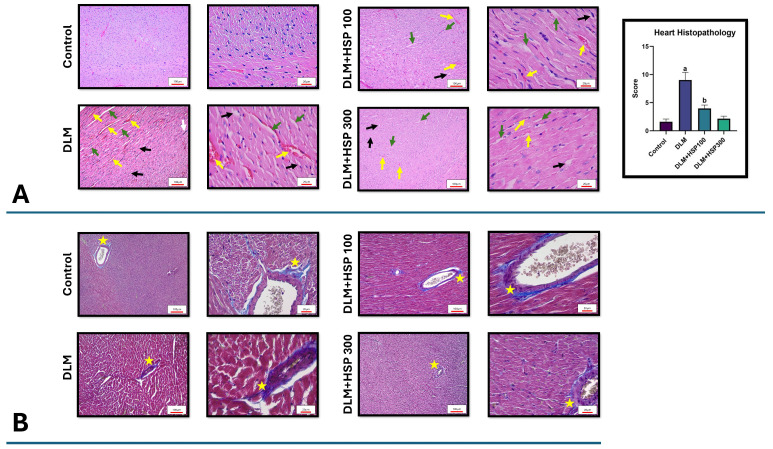
Histopathological evaluation of cardiac tissues obtained from all groups was performed on sections stained with hematoxylin and eosin (H&E) (**A**) and Masson’s trichrome (**B**). In H&E-stained sections, edema in the connective tissue (green arrow), congestion (yellow arrow), inflammatory cell infiltration (white arrow), and vacuolization (black arrow) are indicated. In Masson’s trichrome-stained sections, collagen content in the perivascular areas is demonstrated (yellow star). Statistical significance is denoted as a (*p* < 0.05 vs. control group) and b (*p* < 0.05 vs. DLM group). Each group consisted of 8 animals (*n* = 8).

Histological analysis using H&E staining revealed preserved myocardial architecture in the Control group, characterized by intact striations, branched myofibrils, and continuity between adjacent fibers, with no signs of inflammatory cell infiltration or edema. In contrast, the DLM-treated group exhibited pronounced histopathological alterations, including vacuolization, interstitial edema, congestion, and infiltration of inflammatory cells, indicating significant myocardial injury. HSP administration ameliorated these changes in a dose-dependent manner. The DLM + HSP 100 group demonstrated partial improvement, with reduced inflammatory infiltration and less pronounced edema, whereas the DLM + HSP 300 group exhibited near-complete restoration of normal myocardial architecture (Figure 2A).

Masson’s trichrome staining corroborated these findings by highlighting alterations in collagen deposition. The Control group displayed thin, well-organized collagen fibers primarily localized in perivascular areas. In contrast, the DLM group showed excessive collagen accumulation, with thickened and densely packed fibers indicative of progressive myocardial fibrosis. Treatment with HSP reduced these fibrotic changes, with the DLM + HSP 100 group showing a moderate decrease in collagen density and improved fiber organization, while the DLM + HSP 300 group exhibited collagen distribution closely resembling that of the Control group. Collectively, these results indicate that HSP effectively mitigates DLM-induced structural damage and fibrosis in cardiac tissue, preserving myocardial integrity and reducing pathological remodeling (Figure 2B).

#### 3.2.2. Histological Evaluation of Aortic Tissue (H&E Staining)

**Figure 3:**
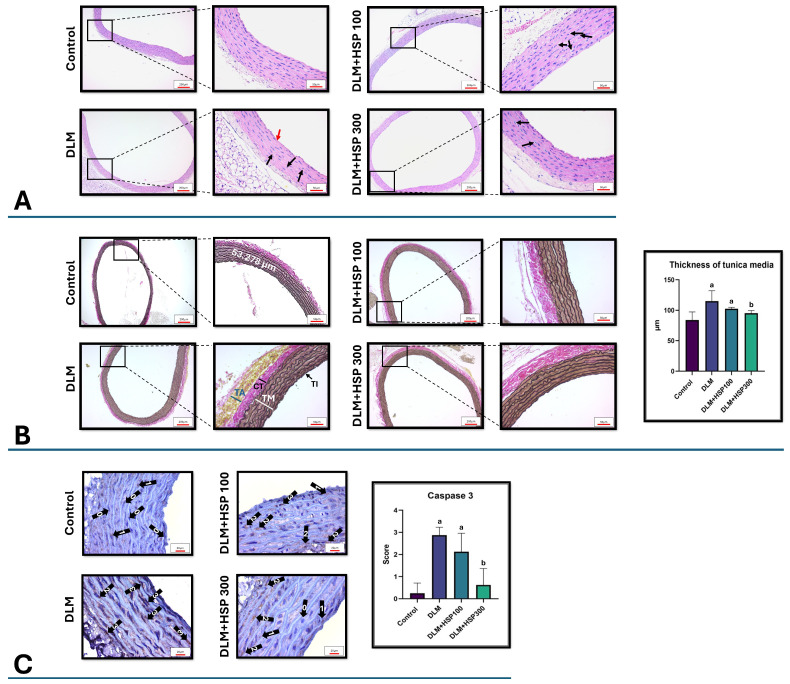
Histopathological and immunohistochemical evaluation of rat aortic tissue sections in all experimental groups. Hematoxylin–Eosin (H&E)-stained sections showing endothelial denudation and intimal damage (red arrow), vacuolization in the tunica media and adventitia (black arrow), and sclerotic changes (**A**). Verhoeff–Van Gieson-stained aortic sections demonstrating the assessment of tunica media thickness. A representative image of tunica media thickness measurement is presented. Measurements were performed using Zen Blue 3.4 software at six randomly selected points along the aorta, and the mean value was calculated for each sample. Abbreviations in the micrographs represent the vascular layers: TA (tunica adventitia), CT (connective tissue), TM (tunica media), and TI (tunica intima). The indicated measurement of 97.188 µm serves as a representative example of the TM thickness quantification (**B**). Immunohistochemical staining of Caspase-3 expression in thoracic aorta tissues. Representative arrows with numerical labels (0, 1, 2, and 3) indicate the staining intensity scores used in the semi-quantitative analysis: 0 (no staining), 1 (weak/mild), 2 (moderate), and 3 (strong staining) (**C**). Statistical significance is denoted by a (*p* < 0.05 vs. control) and b (*p* < 0.05 vs. DLM). Each group consisted of 8 animals (*n* = 8).

H&E staining of aortic sections from the Control group demonstrated well-preserved histological architecture, characterized by a clearly defined tunica intima with intact endothelial lining, a tunica media composed of regularly arranged smooth muscle cells interspersed with elastic fibers, and a structurally continuous tunica adventitia. In contrast, the DLM-treated group exhibited pronounced histopathological alterations, including thickening and sclerotic changes in the aortic wall, fragmentation and atrophy of elastic fibers, loss of endothelial integrity in the intima, and vacuolization within both the media and adventitia, indicative of degenerative and remodeling processes. Additionally, the tunica media displayed disorganized smooth muscle cell alignment and occasional inflammatory cell infiltration, further highlighting vascular injury. Administration of HSP attenuated these DLM-induced alterations in a dose-dependent manner. In the DLM + HSP 100 group, partial preservation of elastic fibers, improved endothelial continuity, and reduced vacuolization were observed. Notably, in the DLM + HSP 300 group, the aortic architecture was largely restored, with well-organized media, intact elastic fibers, and minimal vacuolization, closely resembling the histology of the Control group (Figure 3A). These findings suggest that HSP effectively mitigates DLM-induced structural damage in the aortic wall.

#### 3.2.3. Verhoeff–Van Gieson Staining of Aortic Tissue

Verhoeff–Van Gieson (VVG) staining of aortic sections revealed intact and well-organized elastin fibers in the Control group, with clearly defined wavy patterns within the tunica media, contributing to normal aortic elasticity. In contrast, aortas from the DLM-treated group exhibited marked disruption of elastin fiber architecture, including fragmentation, thinning, and loss of the characteristic wavy pattern. These structural abnormalities were accompanied by a significant thickening of the tunica media, indicative of vascular remodeling and potential loss of elasticity. Quantitative morphometric analysis confirmed that media thickness in the DLM group was significantly increased compared to the Control group. Treatment with HSP ameliorated these histopathological changes in a dose-dependent manner. In the DLM + HSP 100 group, partial preservation of elastin fiber integrity and a moderate reduction in media thickness were observed. Notably, in the DLM + HSP 300 group, the elastin fibers appeared largely continuous and well-organized, and the tunica media thickness was restored to levels comparable to the Control group. Additionally, the connective tissue layer was observed to be thin in the control group, whereas it significantly thickened following DLM administration. This finding indicates that DLM exposure triggers oxidative damage, leading to fibroblast activation and excessive collagen synthesis. The observation that this thickening did not fully revert to its baseline state in the HSP-treated groups suggests that while HSP effectively halted biochemical damage through its antioxidant properties, the experimental timeframe was insufficient for the resolution of the established fibrous tissue (Figure 3B). These findings indicate that HSP effectively protects against DLM-induced elastin fiber disruption and vascular remodeling in the aorta.

#### 3.2.4. Immunohistochemical Staining of Caspase-3 in Aortic Tissue

Immunohistochemical analysis of aortic sections revealed basal caspase-3 expression in the Control group, indicating minimal apoptotic activity under normal conditions. In contrast, the DLM-treated group exhibited a pronounced increase in caspase-3 immunoreactivity, characterized by intense cytoplasmic and nuclear staining within the vascular smooth muscle cells of the tunica media. This observation indicates activation of the apoptotic pathway in response to chronic DLM exposure. In the DLM + HSP 100 group, a moderate reduction in caspase-3 staining was observed, reflecting a partial attenuation of apoptosis; however, the difference did not reach statistical significance compared to the DLM group. Notably, in the DLM + HSP 300 group, caspase-3 expression was markedly reduced, with staining intensity and distribution closely resembling those of the Control group, suggesting near-complete inhibition of DLM-induced apoptotic activity (Figure 3C). These results demonstrate a dose-dependent anti-apoptotic effect of HSP, highlighting its potential in protecting vascular tissues against DLM-induced cellular apoptosis.

## 4. Discussion

There is limited evidence regarding the long-term impact of DLM exposure on cardiovascular tissues, particularly the heart and aorta. In this context, the present study provides novel insights by demonstrating that chronic DLM administration may compromise antioxidant defense mechanisms, enhance inflammatory responses in cardiac tissue, and trigger apoptotic processes in the aorta. Furthermore, our findings suggest that these detrimental alterations could be mitigated by HSP treatment, highlighting its potential as a protective agent against pesticide-induced cardiovascular toxicity. Human exposure to DLM is generally not reported as precise mg/kg doses but rather in terms of plasma/serum concentrations or urinary metabolite levels. Controlled human studies have indicated that the acceptable daily intake for DLM is approximately 0.01 mg/kg [30]. In contrast, acute poisoning cases typically involve substantially higher exposure levels, often exceeding several hundred milligrams, and are associated with pronounced neurotoxic symptoms [18]. Furthermore, estimated daily exposure levels in the general population are reported to be in the µg/kg range [31]. In this context, the dose of 1.25 mg/kg used in the present study is considerably higher than typical environmental or dietary exposure levels, yet remains below those associated with severe acute toxicity in humans. Therefore, this dose does not aim to directly mimic acute human poisoning, but rather represents an experimentally relevant sublethal dose suitable for modeling the potential biochemical and histopathological effects of chronic, cumulative, or subclinical exposure scenarios.

In the present study, chronic DLM exposure induced significant biochemical and histopathological alterations in rats, highlighting the oxidative and inflammatory consequences of prolonged pesticide administration. A marked increase in IMA levels was observed in the DLM-treated group, consistent with previous reports linking elevated IMA to oxidative modifications in albumin structure mediated by ROS [32,33]. Similar clinical observations have been reported in patients with peripheral artery disease and aortic aneurysms, suggesting that DLM promotes oxidative stress, which may contribute to tissue and organ damage [34,35]. Metabolic disturbances were also evident following DLM exposure. Elevated blood glucose levels align with earlier studies indicating that pesticide-induced oxidative stress increases hepatic energy demand, resulting in glycogen depletion and hyperglycemia [36,37]. Regarding lipid metabolism, a non-significant upward trend in TG levels and a slight decrease in HDL were observed in the DLM group. These findings are partially consistent with prior studies reporting variable effects on lipid profiles depending on the type, dose, and duration of pesticide exposure. Notably, total cholesterol levels remained unchanged, contrasting with studies employing higher doses or prolonged exposure [5], underscoring the influence of experimental conditions on the metabolic impact of DLM toxicity. Regarding lipid parameters, our findings revealed a non-significant upward trend in TG levels, in line with studies reporting variability across pesticide models [17,38,39]. Similarly, although a reduction in HDL concentrations was observed in the DLM group, this change was not statistically significant, contrasting with the significant decrease reported by Uchendu et al. 2014 [40]. Moreover, in contrast to Feriani et al., 2016 who reported increased cholesterol levels under higher doses and longer exposure periods, no significant differences in total cholesterol were found in our experimental conditions [5]. These discrepancies emphasize the role of dosage, exposure duration, and experimental setting in determining the metabolic consequences of DLM toxicity. Importantly, the protective role of HSP was evident in our study. Numerous reports highlight the antioxidant potential of flavonoids, particularly HSP, in mitigating oxidative damage and modulating redox homeostasis in various pathological settings [41,42,43]. Consistent with these reports, our data showed that HSP treatment alleviated DLM-induced biochemical alterations, likely through restoration of the oxidant–antioxidant balance. These findings strengthen the hypothesis that HSP may be a promising candidate for reducing oxidative damage in pesticide-induced toxicity models.

Histological examinations further supported the biochemical outcomes. DLM exposure resulted in inflammatory infiltration, myocardial damage, and collagen fiber accumulation, consistent with earlier studies demonstrating pesticide-induced fibrosis and structural remodeling [6,8,44,45,46]. Notably, our Masson’s trichrome results revealed enhanced collagen deposition in the DLM group, indicating progressive fibrotic changes. These alterations may be associated with the release of pro-inflammatory cytokines and extracellular matrix remodeling, which are well-recognized contributors to impaired cardiac function. Importantly, HSP supplementation demonstrated robust protective effects against DLM-induced damage. Previous studies have highlighted HSP’s antioxidant and cytoprotective properties in various cardiovascular models [19,47,48,49]. In line with these findings, HSP treatment in our study mitigated biochemical disruptions, improved oxidant–antioxidant balance, and reduced histopathological alterations, including collagen accumulation and inflammatory infiltration. Similar cardioprotective effects of HSP have been reported in cisplatin- and doxorubicin-induced myocardial injury, as well as in DLM-induced toxicity models [19,49].

Notably, the protective effects were dose-dependent, emphasizing the importance of adequate HSP supplementation for mitigating oxidative and inflammatory damage. Collectively, these findings indicate that chronic DLM exposure exerts multi-faceted cardiotoxic effects, including oxidative stress, metabolic disturbances, inflammation, and fibrotic remodeling. HSP supplementation effectively attenuates these adverse outcomes, supporting its potential as a natural protective agent for preventing or reducing pesticide-induced cardiotoxicity. These results provide a comprehensive basis for future studies evaluating HSP as a protective intervention against chronic pesticide exposure and highlight the relevance of dosage and exposure duration in determining the severity of DLM-induced tissue injury.

Chronic exposure to DLM has been shown to adversely affect the structural integrity of the aorta. Nasuti et al. proposed that pyrethroid-induced free radicals selectively increase aortic wall permeability, resulting in disruption of vascular smooth muscle cell architecture [50]. Similarly, Hachani et al., 2011 reported that pesticide exposure promotes smooth muscle cell dedifferentiation and intimal thickening [51]. Supporting these findings, Feriani et al., 2016 demonstrated that DLM treatment in rats led to lipid droplet accumulation in the arterial intima, endothelial disruption, and medial layer thickening [5]. In line with these observations, our histological analysis revealed sclerotic changes in the aortic wall following DLM exposure, with a significant increase in tunica media thickness. Verhoeff-van Gieson staining further confirmed an increase in the thickness of the tunica media, suggesting vascular remodeling and potential alterations in aortic elasticity. HSP has been recognized for its vascular protective effects, mediated through dual mechanisms: direct scavenging of ROS [52] and enhancement of endogenous antioxidant defenses [53]. Antioxidant compounds have been shown to preserve vascular function under oxidative stress; Jin et al., 2009 demonstrated that apigenin protects against oxidative stress-induced vascular dysfunction [54], while Orallo et al., 2004 reported that HSP exhibits potent vasorelaxant properties [55]. Consistently, our results indicate that high-dose HSP treatment attenuates DLM-induced aortic damage, corroborating findings by Yamamoto et al., 2008 who showed that HSP can reduce vascular hypertrophy [56]. These results support the notion that HSP not only mitigates oxidative stress but also exerts direct vasoprotective effects.

Apoptotic pathways also play a crucial role in DLM-induced vascular injury. Previous studies have shown that DLM generates ROS, causing DNA damage and triggering apoptosis in multiple tissues [57,58,59]. In our study, immunohistochemical analysis revealed significant upregulation of caspase-3 in the aortic tissue of DLM-treated rats, suggesting that p53-mediated activation of caspase-9 subsequently induces caspase-3-dependent apoptosis. These findings are in agreement with prior reports demonstrating DLM-induced caspase-3 activation both in vitro and in vivo, highlighting the contribution of oxidative stress-mediated apoptotic pathways to vascular damage [60,61]. Importantly, HSP supplementation exerted notable anti-apoptotic effects. Kuzu et al., 2021 reported that HSP attenuated sodium arsenite-induced apoptosis by downregulating Bax and caspase-3 expression [43], while Abdelaziz et al., 2020 observed that HSP reduced Bax and increased Bcl-2 levels in methotrexate-induced hepatic toxicity [62]. Similarly, Jia et al., 2022 demonstrated that high-dose HSP significantly decreased cisplatin-induced caspase-3 expression in cardiac tissue [19]. DLM exposure has been shown to rapidly induce the generation of ROS, leading to oxidative stress, lipid peroxidation, and activation of pro-inflammatory and apoptotic pathways shortly after administration. These early molecular events, even within minutes to hours, can initiate cellular dysfunction that contributes cumulatively to chronic structural and functional alterations in cardiovascular tissues [63]. Studies have demonstrated that acute increases in oxidative and inflammatory markers, as well as caspase-mediated apoptosis, occur following DLM exposure, implicating these mechanisms in both immediate and long-term toxicity [64]. Conversely, HSP, a flavonoid with well-documented antioxidant, anti-inflammatory, and anti-apoptotic properties, has been shown to attenuate ROS formation, suppress pro-inflammatory signaling, and reduce caspase activation in various toxicological models. In organophosphate-induced toxicity paradigms, HSP administration significantly reduced oxidative stress and apoptotic markers, supporting its capacity to mitigate early molecular insults [65]. Such evidence suggests that HSP’s inhibition of acute oxidative, inflammatory, and apoptotic responses triggered by DLM may blunt the progression toward chronic cardiovascular damage, providing a mechanistic basis for the observed protective effects in this study.

The existing literature in this field is limited, particularly regarding the long-term effects of chronic DLM exposure on cardiac and aortic tissues. Most studies are restricted to short-term exposures, high-dose applications, or investigations of a single organ system. Similarly, the protective effects of HSP or other flavonoids are generally examined using short-term treatment protocols.

The methodological limitations of the present study include the investigation of only specific DLM doses and exposure durations, the exclusive use of male rat models, and the evaluation of HSP effects at only two doses. Future research should incorporate long-term studies encompassing different doses and exposure durations, experiments that consider both sex and age factors, and investigations into the molecular mechanisms underlying HSP’s protective effects. In our experimental model, the 30-min interval between deltamethrin exposure and hesperidin administration was chosen to evaluate the substance’s ability to mitigate immediate oxidative stress and early-phase apoptotic signaling. While this design demonstrates a strong protective effect, we acknowledge that it does not represent a conventional therapeutic approach where treatment begins after clinical symptoms are fully established. This distinction is crucial for interpreting the clinical translation of our results, and further studies are warranted to explore the efficacy of hesperidin in delayed treatment protocols. Furthermore, a notable limitation of our study is the absence of functional cardiovascular data, such as hemodynamic measurements or heart rate variability. While the histopathological and biochemical findings provide robust evidence of structural damage and oxidative stress, correlating these results with functional impairment would have further strengthened our conclusions.

## 5. Conclusions

Chronic DLM exposure induces multi-faceted cardiovascular toxicity, including oxidative stress, metabolic disturbances, inflammation, fibrotic remodeling, and apoptosis. HSP supplementation effectively mitigates these adverse outcomes, supporting its potential as a natural therapeutic agent against pesticide-induced cardiotoxicity. These findings provide a solid basis for future studies evaluating HSP as a protective intervention against chronic pesticide exposure and highlight the critical role of dose and exposure duration in determining DLM-induced tissue injury severity. Future studies should also compare pre- versus post-treatment strategies to determine whether HSP can prevent or reverse DLM-induced cardiovascular remodeling, further clarifying its potential as both a prophylactic and therapeutic agent.

## Data Availability

The data presented in this study are available on reasonable request from the corresponding author. The data are not publicly available due to institutional and ethical restrictions.

## Abbreviations

The following abbreviations are used in this manuscript:

CHOL	Cholesterol
DLM	Deltamethrin
HDL	High-density lipoprotein
HSP	Hesperidin
IMA	Ischemia-modified albumin
IMT	Intima–media thickness
ROS	Reactive oxygen species
TG	Triglyceride
